# Report from the BV-BRC, CDC, NCBI, and NIAID Viral Sub-Species Classification Workshop

**DOI:** 10.1128/jvi.00210-26

**Published:** 2026-06-12

**Authors:** Elliot J. Lefkowitz, Wiriya Rutvisuttinunt, J. Rodney Brister, Anna Capria, Eleanor S. Click, Jason Caravas, Eneida Hatcher, Duncan MacCannell, Paul Rota, Indresh Singh, Lucy Stewart, Alison St. John, Christian Zmasek, Liliana L. Brown

**Affiliations:** 1 Department of Microbiology, University of Alabama at Birmingham 9968, Birmingham, Alabama, USA; 2 National Institute of Allergy and Infectious Diseases 35037, Rockville, Maryland, USA; 3 National Center for Biotechnology Information, National Library of Medicine, National Institutes of Health 2511, Bethesda, Maryland, USA; 4 J. Craig Venter Institute 272939, La Jolla, California, USA; 5 National Center for Emerging and Zoonotic Infectious Diseases, Centers for Disease Control and Prevention 1242, Atlanta, Georgia, USA; 6 National Center for Immunization and Respiratory Diseases, Centers for Disease Control and Prevention 1242, Atlanta, Georgia, USA; St Jude Children's Research Hospital, Memphis, Tennessee, USA

**Keywords:** viral taxonomy, genomic surveillance, nomenclature, viral sub-species classification

## Abstract

The Viral Sub-Species Classification Workshop, held from 8 to 10 April 2024 at the National Institute of Allergy and Infectious Diseases (NIAID), addressed scientific and technological challenges in the classification of viruses below the level of species. Co-organized by the Bacterial and Viral Bioinformatics Resource Center (BV-BRC), the Centers for Disease Control and Prevention (CDC), the National Center for Biotechnology Information (NCBI), and NIAID, it convened experts in virology, bioinformatics, and public health to discuss topics including tracking the real-time evolution of virus lineages; developing useful and non-stigmatizing nomenclature; supporting global coordination for the development of diagnostics, vaccines, treatments, and public health strategies; and ensuring accurate and timely updates of information. The workshop emphasized the role of a formal viral taxonomy and characterization below the species level, such as clades, genotypes, and lineages. Discussions included systems such as the variants of concern short nomenclature and Pango lineages for respiratory viruses, alongside similar systems for other viral pathogens. Presentations highlighted real-time genomic tracking for timely vaccine updates and addressed challenges in nomenclature systems. Technological advancements and community collaboration were deemed vital. Discussions included high-level descriptions of systems that could be used to track and classify evolving viral lineages; name them using dynamic nomenclature systems; and integrate pathogenic, antigenic, and other phenotypic data with genetic information.

## INTRODUCTION

A vital component of the response to viral outbreaks is the classification and tracking of sub-species-level variation, which is essential for guiding public health risk assessment and communication strategies. Given this rapid and ongoing evolution of viruses, there is a need for sub-species classification to enable timely tracing of pathogen variants with important functional consequences, such as altered antigenicity, pathogenicity, or transmissibility. These sub-species ranks go by many names—including clades, genotypes, and lineages—and are used to monitor viral evolution in real time during outbreaks. Significant scientific and technological challenges remain, including maintaining accurate and timely classification systems during fast-moving outbreaks, developing useful and non-stigmatizing nomenclature for naming lineages, and ensuring global coordination to support diagnostics, vaccines, treatments, and public health strategies.

To address these challenges and prepare for future viral disease outbreaks, the National Institute of Allergy and Infectious Diseases (NIAID)-funded Bacterial and Viral Bioinformatics Resource Center (BV-BRC, hosted by the University of Chicago), the U.S. Centers for Disease Control and Prevention (CDC), the National Center for Biotechnology Information (NCBI), and the NIAID organized a Viral Sub-Species Classification Workshop, held on 8–10 April 2024, at the NIAID conference facilities in Rockville, MD. Workshop details and the agenda are available via BV-BRC (https://www.bv-brc.org/docs/news/2024/2024-04-08-bv-brc-workshop-subspecies.html). Recordings of workshop sessions were made publicly available (https://www.youtube.com/playlist?list=PLWfOyhOW_OasOchpm2zeCvj5AUzo9gFT6). Workshop presenters and their affiliations are listed in Table S1. In addition, participants were encouraged to review the recommended reading list (Reading-List.pdf).

## SUMMARY OF WORKSHOP

Here we present an overview of the discussions and highlight key points identified during the workshop. Across presentations and discussion sessions, participants emphasized the crucial role of sub-species classification in informing outbreak response strategies. Discussions focused on why classification schemes are needed during outbreaks, how they are used, and current gaps in their development, maintenance, and application.

Existing virus classification schemes, such as those for human seasonal influenza A viruses, respiratory syncytial virus, severe acute respiratory syndrome coronavirus 2 (SARS-CoV-2), and human immunodeficiency virus type 1 (HIV-1), help to define: (a) evolving viral lineages and the demarcation criteria used to differentiate them; (b) relevant phenotypic data, such as neutralization and immune resistance patterns; and (c) patterns of segment reassortment or recombination that can drive large-scale phenotypic shifts. Existing classification systems have emphasized integrated phylogenetic approaches. However, classification based on phylogeny is complicated by genetic influx from other species and viral genome fragmentation—and, in some cases, complete excision of genes due to shifts in host selection pressures—that can also yield unique combinations of conserved genes across virus species. More broadly, virus-specific evolutionary processes shape classification and surveillance differently across RNA and DNA viruses. Classification frameworks are therefore not always transferable, as illustrated by swine influenza A H1 and H3, where limited surveillance, cross-species exchange, and the need to integrate genomic, phenotypic, and epidemiological data complicate classification (1, 2).

Given the dynamic nature of viral evolution, the workshop underscored the need for a multifaceted approach to classification that integrates genetic, antigenic, and other phenotypic data to better address public health needs. For example, the genus *Enterovirus* includes multiple species (Enterovirus A, B, C, and D), each containing multiple serotypes with distinct pathogenic properties. As a result, the ICTV classification system—which stops at the rank of species and is based largely on genetic content—does not fully capture the diversity and clinical relevance of variation below the species level. One approach to further integrating phenotypic data into virus classification is through the use of antigenic maps (3), constructed from antibody titers, to support virus classification (4). For instance, H3 influenza virus evolution from 1968 to 2002 progressed through multiple antigenic clusters, contributing to the need for vaccine updates approximately every three years. Incorporating phenotypic criteria when developing classification and naming systems to track sub-species variation could be hugely beneficial to conveying the biological underpinnings of viral lineages.

Different methodological approaches to virus classification were also discussed, including the ICTV’s hierarchical, ranked taxonomic framework and the Pango nomenclature system, which uses hierarchical alphanumeric names (with periods) to define genetically distinct lineages (5, 6). Pango associates lineages with “epidemiological events,” such as geographic importation, phenotypic change, or recombinant/reassortment events. For describing deep historical diversity, Pango-style lineage nomenclature can be particularly useful to track real-time evolution because it supports both fine-scale resolution of hierarchical viral lineages (e.g., SARS-CoV-2) and broader-scale assessments of viral evolution following zoonotic events such as species jumps, recombination, or reassortment (e.g., monkeypox virus [mpox] and influenza viruses).

Existing tools used to explore viral evolution and to generate classification and nomenclature schemes were discussed extensively. There is a clear need for classification systems that can be integrated into a wide range of analytical tools as well as platforms for outbreak assessment and reporting and that support diverse use cases. These tools must also address challenges related to data volume, uneven sampling and representation, and computational requirements. Examples include GISAID-based fitness-ranking tools, Pango lineage nomenclature, and phylogenetic placement tools such as Nextclade (7), UShER (8), Autolin (9), and BV-BRC Placer (10), among others. Their application to SARS-CoV-2, mpox, and influenza—including Pango lineage designation for SARS-CoV-2 and mpox—highlights the need for classification systems that are interoperable, scalable, and supported by sustained surveillance and sequencing. Tables S2 and S3 provide an overview of tools and resources for viral sub-species classification and analysis and summarize several virus classification systems, along with proposed improvements and ongoing challenges. Resources that provide multifaceted search and visualization capabilities for viral sequence data—along with data downloads in customizable formats—are essential for efficient and timely analysis of surveillance results. Similarly, services that offer user-friendly tools and visualizations focused on basic molecular information and associated metadata, as well as targeted outbreak-pathogen information, are critical for a robust outbreak response. However, despite the availability of many bioinformatics resources, addressing complex questions still requires substantial user effort and a deep understanding of both the underlying data and the tools used for analysis.

The workshop presentations showed that successful viral classification systems are developed by collaborative, community-focused approaches. For example, in 2008, the WHO–WOAH–FAO influenza H5N1 Nomenclature Working Group established naming criteria that were implemented globally, becoming the first multilateral consensus nomenclature system for a virus species (11). However, virus-specific approaches to classification and naming often produce systems that are not easily transferable to other viruses. Practical challenges in implementing classification systems may be difficult to overcome. For example, multiple competing classification systems may co-exist, or different groups may assign classifications concurrently that might not necessarily agree. The need for community standards to address classification of viruses discovered through metagenomic sequencing, such as used for waste-water sampling, was also highlighted.

The central challenge is to develop an approach to pathogen surveillance that encompasses genomic data generation and analysis with a lineage classification and nomenclature system that is relatively pathogen agnostic and can be easily and rapidly deployed anywhere it is needed to support monitoring of current and future pathogen threats. Additional challenges include keeping systems current during rapidly evolving outbreaks; preventing names that become too long and unwieldy as lineages continue to diversify over time; agreeing to shared naming approaches to prevent multiple, competing naming systems; and providing centralized resources and accessible reference schemes to define, curate, and update the evolving classification and nomenclature scheme.

Finally, discussions underscored the critical role of genomic surveillance, classification systems, and international collaboration in managing viral disease outbreaks and preparing for future epidemics. Effective responses require systematic characterization of threats and rapid deployment of countermeasures tailored to specific sub-species variants. Genomic surveillance programs face challenges in managing large data sets and maintaining timely communication with public health partners and the public. Nevertheless, state and local public health laboratories remain a core component of the response infrastructure, generating much of the data used to support decision-making at local and national levels. Participants emphasized the importance of active genomic surveillance for prompt and effective response and highlighted the multifactorial nature of infectious disease outbreaks, where virus biology, genomic variation, surveillance, lineage tracking, and epidemiology combine to explain outbreak dynamics and inform response strategies. This is especially relevant for vaccine-preventable infections such as measles.

## KEY CHALLENGES AND FUTURE PERSPECTIVES

### Critical needs

The workshop participants identified key areas where investments would help drive the mission to develop a sensible, usable plan to track the evolution of viral pathogens in real-time during a disease outbreak.

- Capacity building. There is a critical need to build capacity at public health and research laboratories to acquire and use advanced molecular detection methods that assist in early warning for identification of pathogens.
- Sustained funding. Funding follows a panic-neglect cycle; that is, funding peaks during a crisis, then declines once the crisis is resolved, compromising the ability to sustain or update capacity. Unsustained funding prevents efforts to build capacity and capabilities over time.
- Time and effort. Collectively, workshop participants identified staffing and/or resource limitations during outbreaks that limited the available response for efforts such as performing critical assays for pathogen identification. This limited the ability to perform the downstream work needed for pathogen classification and data integration.
- Network/playbook. Workshop participants identified a need and desire to establish a response network and develop a formal preparedness and response plan that would be activated during public health disease-outbreak crises.

- Exercises. The preparedness and response plan will need to be tested using both benchtop and live-action exercises to ensure that the interactive steps performed by the surveillance network during public health crises can be carried out in an efficient, effective manner.

### Real-time genomic tracking and dynamic classification systems

Real-time, worldwide tracking of genomic sequence variants is a foundation of modern-day virus surveillance systems and is critical for an effective response to outbreaks and the timely development and distribution of vaccines and therapeutics. Alongside a surveillance system, the workshop participants discussed the need for adequate classification systems to enable basic research about viral variant characteristics, vaccine and therapeutic development and updates, and public health and scientific communication. Different classification systems, some of which were presented during the workshop along with others that are also in use, may be preferred by particular audiences and organizations and may be more or less suitable, depending on the virus outbreak. Factors like mechanisms of genetic divergence and evolution, status of an outbreak, geographic location and surveillance capability and capacity, and classification systems already in use may all influence the choice of a classification system. Workshop participants discussed at length the needs of classification systems that could integrate information critical to a specific response. For instance, for influenza and SARS-CoV-2, a major effort is to determine whether current vaccines need to be modified to better protect against circulating viral variants. Consequently, a classification system that includes information about antigenicity would be desirable. In other viruses, traits like drug resistance or serotype might be informative, so the chosen classification system would need to be responsive to those needs.

### Clarity and integration of nomenclature systems

In the field of taxonomy, names become a critical point of reference to the taxa into which organisms are classified. During an outbreak, they also provide a point of reference for conversation and action regarding a pathogen and its evolving lineages. Names can also be controversial and harmful due to the use of stigmatizing components in a name, such as geographic location. The workshop highlighted the complexities of naming systems and the necessity for using several complementary layered systems to encompass the different levels of naming resolution needed by the multiple audiences and users of the surveillance data. The participants discussed the benefits of a high-level system that provides easy-to-remember and easy-to-speak names to support the general public’s need for information concerning the severity of major circulating (past or present) lineages, akin to the Greek letter-based system the WHO used for SARS-CoV-2 lineages. Concurrently, a more granular system might better support vaccine efficacy and therapeutic development, where even small genetic differences could result in significant variance in the composition of vaccine or drug candidates. Some participants argued that an intermediate level of granularity that names impactful lineages, but only those with unique disease or epidemiological properties, might be helpful as well. Participants noted that this approach, as implemented by the Pango classification system to name SARS-CoV-2 lineages, continues to be highly successful. An advantage of this approach is that it offers adaptability since the differences and the phenotypic properties that are associated with these genotype-based lineages are defined or discovered post hoc and can be modified as necessary as the characteristics of the virus and resulting disease change.

### Technological choice, advancement, and collaboration

For some viruses, like influenza and SARS-CoV-2, a wide variety of tools have been developed to support virus sequence classification, sample placement on existing phylogenetic trees, and efficient lineage assignment. However, for many viruses, a very limited toolbox exists that limits the effectiveness of a response. Furthermore, participants noted that much of the technology underlying viral classification is supported by very few dedicated individual investigators, many times without any funding support. Workshop participants discussed the benefits of having dedicated efforts and funding to develop and maintain viral classification systems, such that these do not rely exclusively on volunteers or temporary academic positions. In cases where multiple classification systems are being used, it is essential that computational biologists provide mappings that integrate the different approaches used for classification and naming. Support for visualization tools, such as genome and namespace graphs, antigenic maps, phylogenetic trees, and epidemiological tracing for a variety of audiences, is desirable to assist with the understanding of the data and communication. Participants noted the importance of tools being broadly accessible and easy to integrate into common pipelines, not just in places with access to high-performance computing resources but also in low-resource environments. Among the most valuable technologies and resources are common, accurate, up-to-date reference multiple sequence alignments and phylogenetic trees. The phylogenetic trees also serve as an early detection system that may provide an indication of an interesting evolving genotype prior to detection of phenotypic evidence.

### Enhanced data management systems and approaches

Participants discussed data infrastructure needs for efficient viral classification: integration of raw data from a large variety of sequencing efforts; collaboration between the large numbers of groups that analyze the data; the ability to quickly convert data into the various formats required by different analytical tools; ensuring the availability and accessibility of the data and tools in widely diverging resource environments; and the need for both graphically based user interfaces and computationally based application programming interfaces for use by different user communities. These needs, along with many others, are required to establish a robust response infrastructure. Participants discussed the importance of having plans for the establishment, maintenance, governance, and funding sustainability of such systems. Participants also discussed some of the points of consideration when developing a strategy to connect source/sample metadata with virus and lineage names. A wide variety of naming conventions are in use: some approaches, such as those for seasonal influenza virus isolates, include host, geographic, and date information, whereas others use non-descriptive isolate names. However, these short, non-informative names may be preferable if they are connected to publicly available databases with associated well-described metadata.

### Workforce development

Workforce development was a key point of discussion throughout the workshop. Discussions surrounded the establishment of foundational education materials at a multitude of levels (i.e., entry-level employees to senior leadership) and how these materials should be maintained and shared across a network. Considerations echoed by multiple participants included the idea that training materials are most effective when they are shareable, reusable, and audience specific. There is also a need for training tailored to decision-makers and those in leadership positions to provide foundational understanding of virus disease, disease outbreaks, and the need for variant tracking and classification. Participants also noted several challenges such as the lack of a professional society that offers continuing education in outbreak response; the competing responsibilities that arise during an outbreak, especially in resource-challenged areas; the lack of dedicated time for training; and the lack of educational and outreach plans which could help guide workforce development.

## CONCLUDING REMARKS

An effective, life-saving response to a local or worldwide outbreak of infectious disease will be critically dependent on having access to accurate, up-to-date data for pathogen tracking and assessing its genetic and biological characteristics as it spreads. This workshop highlighted the need for robust and adaptable virus classification and nomenclature systems that integrate genetic, pathogenic, ecological, and epidemiological data to support an outbreak response to future emerging viruses. Tracking systems must include robust methods for the collection, analysis, and classification of viral data and provide a multitiered system for naming that serves as a point of reference and discussion of impactful, evolving clades without the use of stigmatizing names. Tool development, technological advancements, educational opportunities, and community collaboration will be vital for the implementation of these systems and ensuring that they are state-of-the-art, useful, and used. These systems must also support the needs of different audiences and resource environments and must be sustainable over time.

The considerations provided in this report will be crucial for sustaining efforts in managing outbreaks of viral disease and preparing for future pandemics. However, further discussions are needed to establish and maintain effective, up-to-date response systems and to define clear governance that is shared among scientists, healthcare providers, public health officials, and regulators charged with this effort.

## SUPPLEMENTAL MATERIAL

Supplemental tables jvi.00210-26-s0001.docx Tables S1 to S3.

ASM does not own the copyrights to Supplemental Material that may be linked to, or accessed through, an article. The authors have granted ASM a non-exclusive, world-wide license to publish the Supplemental Material files. Please contact the corresponding author directly for reuse.
