# Supplementary material for: Report from the BV-BRC, CDC, NCBI, and NIAID Viral Sub-Species Classification Workshop
Source: J Virol. 2026 Jun 12;100(7):e00210-26. doi: 10.1128/jvi.00210-26 (PMC13386822; doi:10.1128/jvi.00210-26)
Supplement: Supplemental tables — Tables S1 to S3. [file jvi.00210-26-s0001.docx]

**SUPPLEMENTAL MATERIALS**

**Table 1: Workshop Speakers**

| **Name** | **Affiliation** |
| --- | --- |
| **Anderson, Tavis** | U.S. Department of Agriculture |
| **Bahl, Justin** | University of Georgia, U.S. |
| **Beck, Andrew** | Division of Viral Diseases, National Center for Immunization and Respiratory Diseases, U.S. Centers for Disease Control and Prevention (CDC) |
| **Brown, Liliana** | NIH/NIAID |
| **Colquhou, Rachel** | University of Edinburgh, U.K. |
| **Donato, Celeste** | Murdoch Children’s Research Institute, Australia |
| **Hatcher, Eneida** | National Center for Biotechnology Information (NCBI), U.S. |
| **Hinrichs, Angie** | University of California Santa Cruz, U.S. |
| **Korber, Bette** | Los Alamos National Laboratory, U.S. |
| **Kuhn, Jens H.** | NIH/NIAID/DCR Integrated Research Facility at Fort Detrick, U.S. |
| **Le Mercier, Philippe** | Swiss-Prot group, Swiss Institute of Bioinformatics, Switzerland |
| **Lefkowitz, Elliot** | University of Alabama at Birmingham (UAB), U.S. |
| **Lewis, Rosamund** | World Health Organization |
| **Neher, Richard** | University of Basel, Swiss Institute of Bioinformatics, Switzerland |
| **O’Toole, Áine** | University of Edinburgh, U.K. |
| **Queen, Krista** | LSU Health Shreveport, U.S. |
| **Roemer, Cornelius** | University of Basel, Swiss Institute of Bioinformatics, Switzerland |
| **Seto, Donald** | George Mason University, U.S. |
| **Simmond, Peter** | University of Oxford, U.K. |
| **Smith, Derek J** | University of Cambridge, U.K. |
| **Wang, Sean** | Minnesota Department of Health, U.S. |
| **Warren, Andrew** | University of Virginia, U.S. |
| **Zmasek, Christian** | J. Craig Venter Institute, U.S. |

For more information about the Workshop, please visit the [BV-BRC Workshop web page](http://bv-brc.org/docs/workshops/2024-bv-brc-workshop-subspecies.html), [Workshop Presentations](https://www.bv-brc.org/docs/workshops/2024-bv-brc-workshop-subspecies.html#agenda-section), [Workshop Recordings](https://www.youtube.com/watch?v=ChAWzKSK0_o&list=PLWfOyhOW_OasOchpm2zeCvj5AUzo9gFT6) and [Additional Discussions and Materials](https://www.reddit.com/r/BRC_users/).

**Table 2: Resources and Tools for Viral Sub-species Classification and Analysis**

| **Names** | **Descriptions** |  | **References** |
| --- | --- | --- | --- |
| **Antigenic Mapping** | Visualization of antigenic differences between virus strains by translating titers into distances, often displayed as spatial maps to understand antigenic evolution. |  | (1). |
| **Autolin** | Automates the identification of new viral lineages by analyzing phylogenetic tree structures and metadata, prioritizing branches based on growth rates and significant mutations. |  | (2) |
| **BV-BRC Archaeopteryx phylogenetic tree viewer** | Visualizing and analyzing phylogenetic trees, offering interactive features to explore and annotate evolutionary relationships. |  | (3) |
| **BV-BRC Subspecies Classification Tools** | Uses reference trees and maximum likelihood placement to assign sequences to specific clades or genotypes. |  | (4) |
| **CoV3D** | A database offering 3D structural information on coronavirus proteins, aiding research into viral mechanisms and treatment development. |  | (5) |
| **CovSPECTRUM** | Designed to track and analyze the genetic diversity and spread of SARS-CoV-2 variants by visualizing mutation frequencies and geographic distribution. |  | (6) |
| **EpiFlu** | GISAID database that facilitates the global sharing of genetic and epidemiological data on influenza viruses, supporting research and surveillance to track virus evolution and inform public health responses. |  | <https://gisaid.org/phylogeny-influenza/>  (7) |
| **GISAID/GISAID Emerging Variants Tracker** | Analyzes mutation constellations in sequences, ranking variants based on epidemiological risk, spread, and acceleration. |  | (8) |
| **HIV Databases** | Exploration and analysis of integrated global HIV sequence and immunological data |  | (9)  (10) |
| **International Committee on Taxonomy of Viruses (ICTV)** | Classifies and names viruses based on evolutionary relationships, genetic similarity, and common phenotypic criteria. Uses virus family-specific Study groups to generate and review proposals for taxonomic updates prior to membership ratification. |  | [https://ictv.global](https://ictv.global/)  (11) |
| **Multiple Sequence Alignment Viewer (MSA Viewer)** | Analyze and visualize the alignment of multiple genetic sequences, aiding in the identification of conserved regions and evolutionary relationships. |  | (12) |
| **National Center for Biotechnology Information (NCBI) Virus** | Data and information resource offering virus sequence data, flexible data search interfaces, download, and visualization at no cost and without need for registration. Integrates data from GenBank, BioProject, BioSample, SRA, and other related resources. |  | <https://www.ncbi.nlm.nih.gov/labs/virus> |
| **Nextclade** | A component of Nextstrain, Nextclade analyzes SARS-CoV-2 sequences to determine clade assignments and assess mutations relative to reference clades. |  | (13) |
| **Nextstrain** | A platform that visualizes the real-time evolution and spreads of pathogens, including viruses, by integrating genomic data into interactive phylogenetic trees. |  | (14) |
| **Pangolin** | Software tool used to assign SARS-CoV-2 sequences to pango lineages, helping researchers track the virus's evolution and spread by providing detailed lineage information. |  | (15) |
| **Pango Nomenclature Tool** | Provides a hierarchical system for lineage determination, reflecting evolutionary history with unique alphanumeric codes. |  | (16) |
| **PASC (Pairwise Sequence Comparison)** | Used for filovirus classification, focusing on average nucleotide identity for species and clade demarcation. |  | (17) |
| **phyloP** | A tool that measures evolutionary conservation at individual nucleotide positions, helping identify regions under selective pressure across multiple species. |  | (18) |
| **Rotavirus Classification Tools** | Online tools using random forest approaches and tree phylogeny for classifying rotavirus into groups A, B, and C. |  | (19) |
| **Treenome** **Browser** | Exploration of large phylogenetic trees and multiple sequence alignments, facilitating the understanding of complex evolutionary relationships. |  | (20) |
| **Taxonium** | Visualizing and navigating large phylogenetic trees (e.g., UShER), enabling users to efficiently explore and analyze evolutionary relationships among vast numbers of sequences. |  | (21) |
| **UniProt/UniProtKB** | Offers a protein sequence and functional information database with detailed annotations on protein characteristics, functions, and interactions across various organisms. |  | (22) |
| **UShER** | Ultra-fast sample placement in existing phylogenetic trees, maintaining large SARS-CoV-2 trees with incremental updates. |  | (23) |
| **ViralZone** | Provides comprehensive information on virus families, including their structure, life cycle, and host interactions, to support virology research and education. |  | (24) |
| **Wasabi** | A web-based tool for visualizing and interacting with large phylogenetic trees, allowing users to explore evolutionary relationships and annotate tree data dynamically. |  | (25) |
| **World Health Organization/World Organization for Animal Health/Food and Agriculture Organization (WHO-WOAH-FAO) H5N1 Nomenclature Working Group** | An international collaboration that standardizes the naming and classification of H5N1 influenza viruses, integrating genetic data to address concerns about stigmatization and improve global communication. |  | (26) |

**REFERENCES**

1. Smith DJ, Lapedes AS, de Jong JC, Bestebroer TM, Rimmelzwaan GF, Osterhaus AD, Fouchier RA. 2004. Mapping the antigenic and genetic evolution of influenza virus. Science 305:371–6.

2. McBroome J, de Bernardi Schneider A, Roemer C, Wolfinger MT, Hinrichs AS, O'Toole AN, Ruis C, Turakhia Y, Rambaut A, Corbett-Detig R. 2024. A framework for automated scalable designation of viral pathogen lineages from genomic data. Nat Microbiol 9:550–560.

3. Zmasek CM, Eddy SR. 2001. ATV: display and manipulation of annotated phylogenetic trees. Bioinformatics 17:383–4.

4. Olson RD, Assaf R, Brettin T, Conrad N, Cucinell C, Davis JJ, Dempsey DM, Dickerman A, Dietrich EM, Kenyon RW, Kuscuoglu M, Lefkowitz EJ, Lu J, Machi D, Macken C, Mao C, Niewiadomska A, Nguyen M, Olsen GJ, Overbeek JC, Parrello B, Parrello V, Porter JS, Pusch GD, Shukla M, Singh I, Stewart L, Tan G, Thomas C, VanOeffelen M, Vonstein V, Wallace ZS, Warren AS, Wattam AR, Xia F, Yoo H, Zhang Y, Zmasek CM, Scheuermann RH, Stevens RL. 2023. Introducing the Bacterial and Viral Bioinformatics Resource Center (BV-BRC): a resource combining PATRIC, IRD and ViPR. Nucleic Acids Res 51:D678–D689.

5. Gowthaman R, Guest JD, Yin R, Adolf-Bryfogle J, Schief WR, Pierce BG. 2021. CoV3D: a database of high resolution coronavirus protein structures. Nucleic Acids Res 49:D282–D287.

6. Chen C, Nadeau S, Yared M, Voinov P, Xie N, Roemer C, Stadler T. 2022. CoV-Spectrum: analysis of globally shared SARS-CoV-2 data to identify and characterize new variants. Bioinformatics 38:1735–1737.

7. Shu Y, McCauley J. 2017. GISAID: Global initiative on sharing all influenza data - from vision to reality. Euro Surveill 22.

8. Elbe S, Buckland-Merrett G. 2017. Data, disease and diplomacy: GISAID's innovative contribution to global health. Glob Chall 1:33–46.

9. Gaschen B, Kuiken C, Korber B, Foley B. 2001. Retrieval and on-the-fly alignment of sequence fragments from the HIV database. Bioinformatics 17:415–8.

10. Yoon H, Macke J, West AP, Jr., Foley B, Bjorkman PJ, Korber B, Yusim K. 2015. CATNAP: a tool to compile, analyze and tally neutralizing antibody panels. Nucleic Acids Res 43:W213–9.

11. Lefkowitz EJ, Dempsey DM, Hendrickson RC, Orton RJ, Siddell SG, Smith DB. 2018. Virus taxonomy: the database of the International Committee on Taxonomy of Viruses (ICTV). Nucleic Acids Res 46:D708–D717.

12. Anderson CL, Strope CL, Moriyama EN. 2011. SuiteMSA: visual tools for multiple sequence alignment comparison and molecular sequence simulation. BMC Bioinformatics 12:184.

13. Aksamentov IR, C; Hodcroft, EB; Neher, R.A. 2021. Nextclade: clade assignment, mutation calling and quality control for viral genomes. Journal of Open Source Software 6.

14. Hadfield J, Megill C, Bell SM, Huddleston J, Potter B, Callender C, Sagulenko P, Bedford T, Neher RA. 2018. Nextstrain: real-time tracking of pathogen evolution. Bioinformatics 34:4121–4123.

15. de Bernardi Schneider A, Su M, Hinrichs AS, Wang J, Amin H, Bell J, Wadford DA, O'Toole A, Scher E, Perry MD, Turakhia Y, De Maio N, Hughes S, Corbett-Detig R. 2024. SARS-CoV-2 lineage assignments using phylogenetic placement/UShER are superior to pangoLEARN machine-learning method. Virus Evol 10:vead085.

16. Rambaut A, Holmes EC, O'Toole A, Hill V, McCrone JT, Ruis C, du Plessis L, Pybus OG. 2020. A dynamic nomenclature proposal for SARS-CoV-2 lineages to assist genomic epidemiology. Nat Microbiol 5:1403–1407.

17. Bao Y, Chetvernin V, Tatusova T. 2012. PAirwise Sequence Comparison (PASC) and its application in the classification of filoviruses. Viruses 4:1318–27.

18. Pollard KS, Hubisz MJ, Rosenbloom KR, Siepel A. 2010. Detection of nonneutral substitution rates on mammalian phylogenies. Genome Res 20:110–21.

19. Tran H, Friendship R, Poljak Z. 2023. Classification of group A rotavirus VP7 and VP4 genotypes using random forest. Front Genet 14:1029185.

20. Kramer AM, Sanderson T, Corbett-Detig R. 2023. Treenome Browser: co-visualization of enormous phylogenies and millions of genomes. Bioinformatics 39.

21. Sanderson T. 2022. Taxonium, a web-based tool for exploring large phylogenetic trees. Elife 11.

22. UniProt C. 2025. UniProt: the Universal Protein Knowledgebase in 2025. Nucleic Acids Res 53:D609–D617.

23. Turakhia Y, Thornlow B, Hinrichs AS, De Maio N, Gozashti L, Lanfear R, Haussler D, Corbett-Detig R. 2021. Ultrafast Sample placement on Existing tRees (UShER) enables real-time phylogenetics for the SARS-CoV-2 pandemic. Nat Genet 53:809–816.

24. De Castro E, Hulo C, Masson P, Auchincloss A, Bridge A, Le Mercier P. 2024. ViralZone 2024 provides higher-resolution images and advanced virus-specific resources. Nucleic Acids Res 52:D817–D821.

25. Veidenberg A, Medlar A, Loytynoja A. 2016. Wasabi: An Integrated Platform for Evolutionary Sequence Analysis and Data Visualization. Mol Biol Evol 33:1126–30.

26. World Health Organization/World Organisation for Animal HF, Agriculture Organization HNEWG. 2014. Revised and updated nomenclature for highly pathogenic avian influenza A (H5N1) viruses. Influenza Other Respir Viruses 8:384–8.

**Table 3: Summary of Virus Classification Systems, Proposed Improvements, and Challenges**

| **Virus** | **Current System** | **Challenges** | **Proposed Improvements** |
| --- | --- | --- | --- |
| Adenoviruses | Classified into species (A-G) and serotypes based on biological properties, neutralization assays, and genomic sequencing of specific regions. | High genetic diversity, frequent recombination events, resource-intensive manual curation, lack of comprehensive tools. | Integrating whole-genome sequencing for more detailed classification, developing automated and real-time classification tools, and standardized nomenclature system. |
| Filoviruses | Relies on phylogenetic analysis, PASC tools for average nucleotide identity, and type viruses as reference strains. | High genetic diversity, complex and resource-intensive classification, high pathogenicity, biocontainment requirements, standardized naming conventions, and integrating diverse data sources. | Developing a dynamic, real-time classification system with automated updates, standardized nomenclature, integrating genetic, phenotypic, and epidemiological data. |
| HIV-1 | Classified into various subtypes and circulating recombinant forms, focusing on genetic and antigenic diversification. | Vast genetic diversity, rapid mutation rate, complicates vaccine and treatment development, resource-intensive monitoring, integrating genetic and antigenic data, ethical considerations in naming. | Enhanced tracking of recurrent mutations and their phenotypic implications. |
| Influenza Virus | Classified using Hemagglutinin (HA) and Neuraminidase (NA) genes, with real-time tracking for vaccine updates. | Rapid evolution and frequent antigenic drift, complex and slow-to-update nomenclature, integrating diverse data sources, maintaining centralized references, balancing detailed classification with clear communication. | Adoption of a pango-style letter.number.number pattern for new clades, suggested by algorithms and manually picked. |
| MPOX | Two distinct clades based on genotypic and phenotypic characteristics and geographic location. | Genetic diversity, significant phenotypic differences, lack of strong temporal features, long phylogenetic branches, integrating genetic, phenotypic, and epidemiological data, ethical considerations in naming. | More granular classification to track genetic diversity and phenotypic changes. |
| Rotaviruses | Classified based on the VP6 gene, further classified into G (VP7) and P (VP4) types, relying on manual curation and expert input. | High genetic diversity, frequent reassortment, resource-intensive manual curation, lack of comprehensive tools. | Developing automated, real-time classification tools, integrating genetic and antigenic data, standardized nomenclature system. |
| RSV | Based on phylogenetic information and antigenic data. | Genetic diversity, need for effective classification systems, rapid evolution, integrating genetic and antigenic data, lack of standardized nomenclature, resource-intensive updates. | Adoption of a pango-style nomenclature for better tracking and classification. |
| SARS-CoV-2 | Pango lineage nomenclature, phylogenetically based, highlights epidemiologically relevant events. | Rapid evolution, frequent mutations, recombination events, complex pango nomenclature system, integrating diverse data sources, ethical considerations in naming, sustainable genomic surveillance. | Use of short names alongside long names for better retraceability, tracking highly recurrent mutations, defining lineages based on significant phenotypic changes. |
